# Preclassification of Broadband and Sparse Infrared Data by Multiplicative Signal Correction Approach

**DOI:** 10.3390/molecules27072298

**Published:** 2022-04-01

**Authors:** Hafeez Ur Rehman, Valeria Tafintseva, Boris Zimmermann, Johanne Heitmann Solheim, Vesa Virtanen, Rubina Shaikh, Ervin Nippolainen, Isaac Afara, Simo Saarakkala, Lassi Rieppo, Patrick Krebs, Polina Fomina, Boris Mizaikoff, Achim Kohler

**Affiliations:** 1 Faculty of Science and Technology, Norwegian University of Life Sciences, 1430 Ås, Norway; valeria.tafintseva@nmbu.no (V.T.); boris.zimmermann@nmbu.no (B.Z.); johanne.heitmann.solheim@nmbu.no (J.H.S.); achim.kohler@nmbu.no (A.K.); 2 Research Unit of Medical Imaging, Physics and Technology, Faculty of Medicine, University of Oulu, 90570 Oulu, Finland; vesa.k.virtanen@oulu.fi (V.V.); simo.saarakkala@oulu.fi (S.S.); lassi.rieppo@oulu.fi (L.R.); 3 Department of Applied Physics, University of Eastern Finland, 70210 Kuopio, Finland; rubina.shaikh@uef.fi (R.S.); ervin.nippolainen@uef.fi (E.N.); isaac.afara@uef.fi (I.A.); 4 Department of Orthopedics, Traumatology, Hand Surgery, Kuopio University Hospital, 70210 Kuopio, Finland; 5 Institute of Analytical and Bioanalytical Chemistry, Ulm University, 89081 Ulm, Germany; patrick.krebs@uni-ulm.de (P.K.); polina.fomina@uni-ulm.de (P.F.); boris.mizaikoff@uni-ulm.de (B.M.)

**Keywords:** water spectrum, sparse spectra, quality spectra, spectral preclassification, PCA, quantum cascade lasers, OPUS

## Abstract

Preclassification of raw infrared spectra has often been neglected in scientific literature. Separating spectra of low spectral quality, due to low signal-to-noise ratio, presence of artifacts, and low analyte presence, is crucial for accurate model development. Furthermore, it is very important for sparse data, where it becomes challenging to visually inspect spectra of different natures. Hence, a preclassification approach to separate infrared spectra for sparse data is needed. In this study, we propose a preclassification approach based on Multiplicative Signal Correction (MSC). The MSC approach was applied on human and the bovine knee cartilage broadband Fourier Transform Infrared (FTIR) spectra and on a sparse data subset comprising of only seven wavelengths. The goal of the preclassification was to separate spectra with analyte-rich signals (i.e., cartilage) from spectra with analyte-poor (and high-matrix) signals (i.e., water). The human datasets 1 and 2 contained 814 and 815 spectra, while the bovine dataset contained 396 spectra. A pure water spectrum was used as a reference spectrum in the MSC approach. A threshold for the root mean square error (RMSE) was used to separate cartilage from water spectra for broadband and the sparse spectral data. Additionally, standard noise-to-ratio and principle component analysis were applied on broadband spectra. The fully automated MSC preclassification approach, using water as reference spectrum, performed as well as the manual visual inspection. Moreover, it enabled not only separation of cartilage from water spectra in broadband spectral datasets, but also in sparse datasets where manual visual inspection cannot be applied.

## 1. Introduction

Infrared spectroscopy has been widely used in bio-medicine, pharmacology, chemistry, and other fields for chemical analysis of intact materials [1,2,3]. Particularly, the use of infrared spectroscopy combined with multivariate data analysis is gaining popularity as a rapid diagnostic method to distinguish between healthy and diseased tissues or cells in medical applications, such as cancer identification and early stage cartilage degradation [4,5]. Since infrared spectroscopy can simultaneously measure multiple biochemical components, such as collagen, proteoglycans, and water, it has high potential in diagnostics of various compositional changes that occur in articular cartilage due to osteoarthritis and other joint diseases [6,7,8,9,10,11,12].

Fourier transform infrared spectroscopy (FTIR) in the mid–infrared (MIR) region of the electromagnetic spectrum (400–4000 cm${}^{-1}$), combined with microspectroscopy, has been applied for detecting diseases in cartilage and bone tissues [13], pathogenesis and repair of cartilage [14], and osteoarthritic cartilage changes in a rabbit model [10,15,16]. Similarly, near-infrared (NIR) spectroscopy (spectral range (4000–10,000 cm${}^{-1}$) has been successfully used in diagnosis for different kinds of cartilage and bone disease, such as assessment of the matrix constituents of cartilage [17,18], in vivo arthroscopic monitoring of cartilage defects [19], and non-destructive evaluation of articular cartilage [20,21,22]. Furthermore, infrared fiber optic probes coupled with FTIR and NIR spectrometers have shown great success in clinical studies [11,23,24,25,26,27,28].

For in vivo assessment of cartilage tissue condition with infrared spectroscopy, it is crucial to identify water content, collagen, and proteoglycan content [29]. It is pivotal that the measurement is quick, since the surgeon can keep the probe at a defined position of the cartilage only for a short time. There are two primary reasons for a short acquisition time. First, there is an overall time limitation for knee arthroscopy. Nowadays, the total time for knee arthroscopy is about 30 min for each patient, including 25 min of repairs and 5 min for diagnosis. In addition, it is very difficult for the surgeon to operate through the small hole that is available during surgery. Therefore, it is not easy to keep the probe steady and fixed at one position for a longer time. According to the experience of surgeons who use NIR probes in vivo, a max time for fixation of the probe is about 10 s.

To achieve sufficient signal-to-noise ratio during a short acquisition time, quantum cascade lasers (QCLs) can be used as radiation sources. The development of QCLs caused a breakthrough in the field of spectroscopy, allowing much faster measurements of samples due to increased spectral power density when compared to global infrared (IR) light sources employed in FTIR spectrometers [30]. Two types of QCLs have been developed: Tunable QCLs and QCLs with fixed wavelengths. Typical tunable QCLs cover relatively limited ranges of spectral frequencies (approximately 20–200 cm${}^{-1}$, depending on spectral resolution). The QCLs with fixed frequencies are very sharp and cheap compared to tunable QCLs. Furthermore, tunable QCLs have limitations with the stability and laser intensity variations, and low tuning range prevents from monitoring complex species [31,32]. While previous ex vivo studies mostly utilized the entire mid-infrared spectrum to get qualitative and quantitative information about the chemical composition of cartilage samples [11,12,15], with QCLs only limited regions in the infrared spectral range can be measured. In the ongoing Horizon 2020 Photonics21 project MIRACLE [33,34], an arthroscopic IR fiber-optic probe coupled to a spectrometer operating seven MIR QCLs with fixed wavelengths is being developed [6,35,36].The goal is to use the probe as an in vivo diagnostic tool for analysing degeneracy status of articular cartilage during arthroscopy surgery.

In sparse spectral measurements, such as the ones conducted with QCLs with fixed wavelengths, the choice of measurement wavelengths is based on maximization of diagnostic value achieved regarding the application that is being addressed. To obtain that, and at the same time to keep the end price of the product in a reasonable range, seven wavenumbers (7 WNs) for QCLs were selected in the project MIRACLE. The seven QCL WNs covered not only the diagnostic absorption signatures of articular cartilage, but they also allowed standardization of the acquired signals regarding baseline and signal intensity variations (i.e., additive and multiplicative effects) [31,37].

During in vivo measurements, variation in probe contact with the articular cartilage surface can affect the quality of the measured data. As low quality spectra we refer to spectra that have either low total signal or a high water signal and low cartilage signal. A high water signal and low cartilage signal appear when the contact of the probe with the cartilage is insufficient and the ratio of signals belonging to cartilage and matrix surrounding the cartilage (i.e., water-rich synovial fluid) can be skewed in favor of the matrix. This can result with spectra with low analyte (cartilage) signals containing mostly matrix signals. Such spectra do not contain any diagnostics value for the cartilage quality. Hence, a robust preclassification approach which can classify entire spectra that contain cartilage and water/low cartilage signal is needed. It is important to stress that proposed preclassification in this study is different than the standard quality test (QT) of spectral data presented in the literature. The most common approaches of QT of spectral data are based on noise in the spectra, signal-to-noise ratio, peak intensities, baseline variation, signal intensity (both too low and too high), signal fringes, distortions and artifacts (for example, due to radiation scattering), and chemical interference (H${}_{2}$O, CO${}_{2}$) [38,39,40]. In the process of QT, the spectra containing high level of noise and/or too low signal-to-noise are removed. Spectra with low intensity of pre-selected relevant peaks, such as amide I, or spectra with high intensity of interfering chemicals, such as water vapor, carbon dioxide or paraffin, are being removed as well. Most of these QT approaches are based on in-house build routines and on operator experience. Several methods are provided by spectrometer producers as a part of their spectra acquisition and analysis software, such as OPUS (Bruker Optik GmbH), and OMNIC (Thermo Scientific). However, since such quality tests are often developed for specific applications, they cannot be applied universally. For example, OPUS quality test has been originally developed for FTIR measurements of bacteria, and it is sub-optimal for measurements of other types of microorganisms, such as yeasts and filamentous fungi [41].

For in vivo measurements, visual inspection of spectra needs to be replaced by automatic preclassification. The existing quality checks are either insufficient and/or based on broad spectral regions, and they cannot be applied on sparse datasets. Thus, an approach is needed that can preclassify sparse spectra into spectra with high analyte signals (cartilage spectra) and low analyte signals (water or low absorbance spectra). The aim of this paper was therefore to develop a viable approach for assessing sparse spectral data, and it was based on sparse data with the seven WNs as chosen in the Miracle project [33,34].

## 2. Results

### 2.1. Broadband FTIR-ATR Spectra of Cartilage Samples

**Figure 1:**
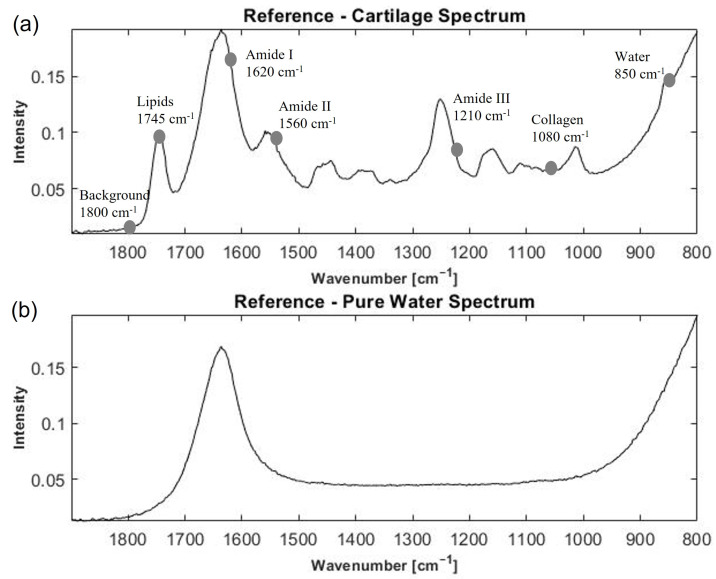
Examples of water and cartilage spectra. (**a**) Cartilage spectrum from dataset 1 (human), (**b**) Pure water spectrum.

**Figure 2:**
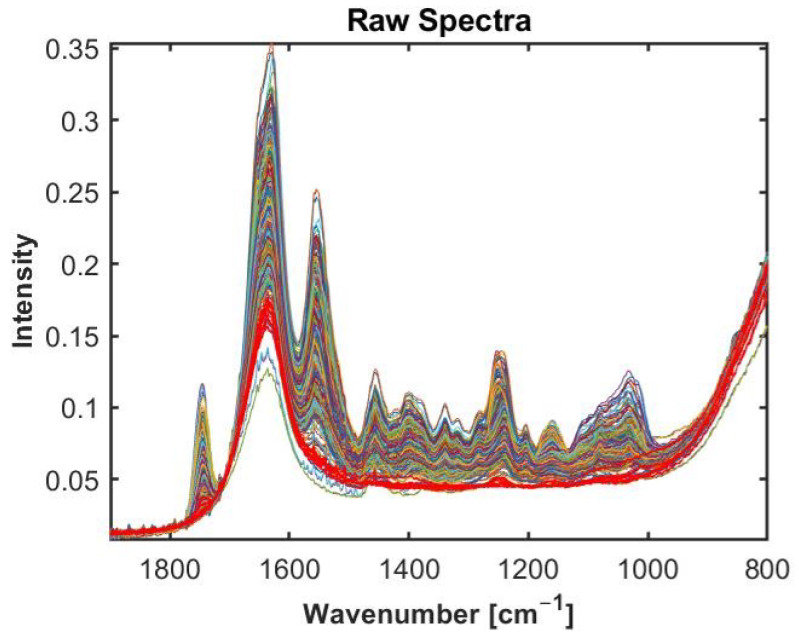
Raw spectra of Human dataset in range from 800 to 1900 cm${}^{-1}$. Highlighted spectra in red color represent water/analyte-poor spectra, whereas the remaining spectra are analyte-rich (cartilage) spectra.

For comparison, a nearly pure cartilage and water spectrum are shown in Figure 1. The absorption bands of amide I (1600–1700 cm${}^{-1}$), amide II (1500–1600 cm${}^{-1}$), amide III (1200–1300 cm${}^{-1}$), and lipids (1745 cm${}^{-1}$) dominate the spectral profile. There is a strong peak of the C-N stretch and N-H bend vibration at 1560 cm${}^{-1}$ linked to amide II [10], the C=O vibration at 1620 cm${}^{-1}$ represent amide I [10,42], and the peak at 1080 cm${}^{-1}$ is the C-O stretch of the carbohydrate residues in collagen and proteoglycans [43]. Conversely, the water spectrum displays a flat spectral signature almost everywhere in the fingerprint region, except for broad peaks at around 1630 and 800 cm${}^{-1}$ (see Figure 1b). In Figure 2 the raw spectra of dataset 1 (human samples measured by Alpha II FTIR spectrometer (Bruker Optics, Ettlingen, Germany)), equipped with a globar MIR source coupled to a single reflection diamond attenuated total reflection (ATR) accessory Platinum (Bruker Optics, Ettlingen, Germany) are shown. Some of the spectra show clear cartilage signals, while some of the spectra contain almost no signals of cartilage and are dominated by water signals (highlighted in red colour). Such spectra do not contain enough of the analyte (i.e., cartilage) information in order to be used for cartilage quality assessment.

The visual identification of the water spectra and spectra containing cartilage specific signals seems to be easily performed even by visual inspection when we have broadband spectra Figure 2. However, performing visual inspection of the sparse spectra is challenging. Visual inspection is also not suitable as a diagnostic tool during surgery. Therefore, there is need for an automated method that can work both for broadband and sparse data. Below, we will evaluate whether a water or cartilage reference spectrum is more suitable for preclassification.

### 2.2. Preclassification Based on Broadband Spectra

**Figure 3:**
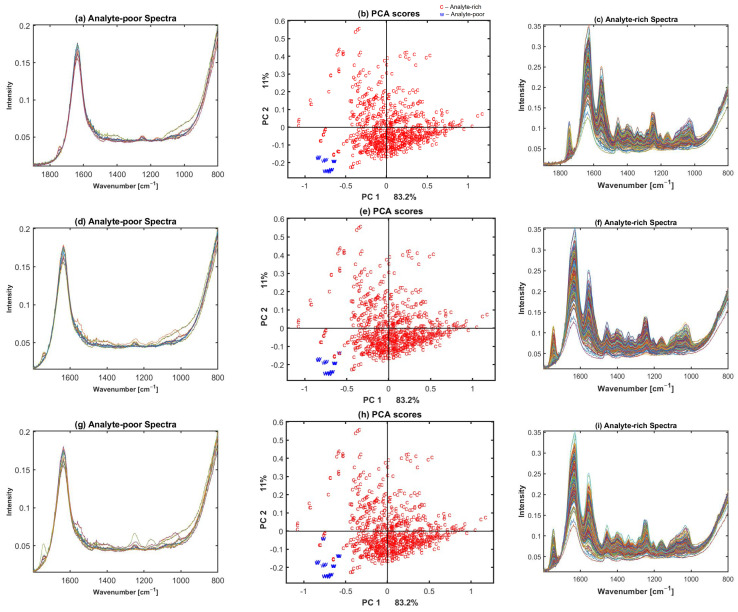
Results of preclassification using dataset 1 broadband spectra. (**a**,**d**,**g**) Water/low absorbance (analyte-poor) spectra and (**c**,**f**,**i**) cartilage/analyte-rich spectra identified by the MSC approach with water spectrum as reference. (**b**,**e**,**h**) PCA scores obtained by different RMSE thresholds, 0.13 for (**b**), 0.144 for (**c**), and 0.152 for (**h**). c and w in PCA scores correspond to cartilage and water spectrum.

In the proposed Multiple Signal Correction (MSC) based preclassification method, we suggest using water spectrum as a reference in the MSC model. Figure 3 shows the results of preclassification when a spectrum of water sample was measured and used in the algorithm. For this dataset, an optimal θ was found to be 0.13 for the broadband dataset 1 (human dataset measured with Alpha II FTIR spectrometer). This selection of the threshold was based on the visual inspection of the spectra. Thus, if a spectrum had an RMSE higher than the threshold RMSE(ϵ)>θ=0.13, the algorithm assigned the spectrum to be an analyte-rich (cartilage) spectrum, while otherwise it was identified as an analyte-poor (water or low absorbance) spectrum. We can see in Figure 3 that water—spectra with low cartilage signals—is correctly separated from the cartilage spectra (see Figure 3a,c,d,f,g,i). From a total of 814 spectra in dataset 1, 21 spectra were identified as water—low cartilage signal spectra. The annotations performed using the PCA score plots in Figure 3b,e,h support the selection of optimal thresholds. We can clearly see a group of water spectra or analyte-poor spectra in blue, and when the threshold θ is increased, some cartilage or analyte-rich spectra were identified as water spectra (see also Supplementary Material Figures S2 and S3 for datasets 2 and 3). The PCA was done on raw spectra without any pre-processing.

**Figure 4:**
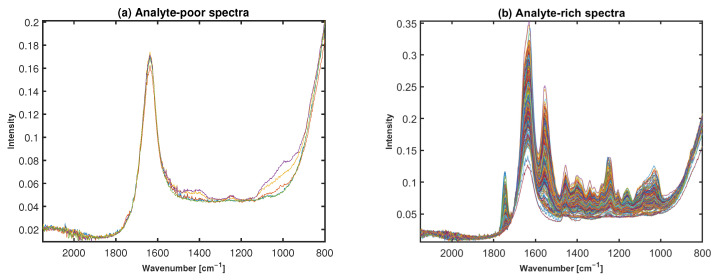
Visual presentation of broad band spectrum after using signal-to-noise ratio (**a**) Water/Analyte-poor Spectra (**b**) Cartilage/Analyte-rich Spectra.

In addition to PCA, an annotation by considering the signal-to-noise ratio was used, Signal/Noise, where signal represents the absorbance signal in the region 920–1200 cm${}^{-1}$ related to polysaccharide (chemical), and the noise is calculated in the region 2000–2100 cm${}^{-1}$. Almost the same spectra were annotated by both PCA analysis and Signal/Noise and these spectra were correctly classified by the MSC preclassification algorithm; spectra separation can be seen for Signal/Noise in Figure 4.

Another alternative for the preclassification algorithm which was also tested in this study is to use a cartilage spectrum as a reference in MSC. Naturally, the conditions for the water and cartilage spectra classification will be the opposite of what is given in Equation (4). We tested different cartilage spectra containing different shares of cartilage information (e.g., collagen, amide I and II) as reference spectra in addition to the representative one presented in Figure 1a. We also tested different RMSE thresholds θ in the algorithm. However, the MSC model did not provide clear separation of water from cartilage spectra irrespective of the reference and thresholds used. Some results are presented in Figure S1. We can see that a number of cartilage spectra were classified as water spectra.

Proposed MSC preclassification approach was performed on dataset 2 (human data measured by Alpha II FTIR spectrometer (Bruker Optics, Ettlingen, Germany), equipped with a deuterated triglyceride sulphate (DTGS) detector, coupled to a single reflection diamond attenuated total reflection (ATR) accessory Platinum (Bruker Optics, Ettlingen, Germany)) and three (bovine) spectral data. The results of MSC preclassification applied to dataset 2 and dataset 3 samples are provided in Supplementary Material Figures S2 and S3. For dataset 2, out of total 815 spectra, six spectra are identified as water. The same spectra were identified for the sparse seven WN spectra of dataset 2. The optimal RMSE threshold for broadband was 0.12, while the RMSE threshold of 0.012 was optimal for seven WN spectra. MSC preclassification on dataset 3 gave nine spectra identified as water out of 396 spectra. The optimal RMSE threshold of 0.5 was found for the broadband dataset and an RMSE threshold of 0.015 was optimal for the seven WN spectral dataset.

### 2.3. Preclassification Based on Laser Wavelengths

The same algorithm but with an RMSE threshold θ=0.42 was tested on the sparse seven WN data.

**Figure 5:**
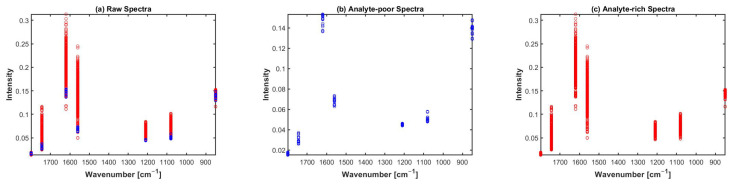
Visual presentation of Human spectral data (set 2) 7 WNs after using MSC preclassification approach, (**a**) raw spectra, (**b**) water/analyte-poor spectra, and (**c**) cartilage/analyte-rich spectra.

Figure 5 shows the sparse spectral data corresponding to the laser wavelengths of the Miracle probe. As can be seen, the sparse data make it visually very hard to identify water and cartilage spectra, while water spectra and cartilage spectra were easily identifiable in Figure 2 of the broadband spectra. Therefore, the visual inspection is not possible when sparse data are used.

As for the broadband spectra, a pure water spectrum was used as a reference spectrum in the MSC model to obtain the preclassification. Seven wavenumbers were used and a threshold θ was adjusted. For the sparse data, an optimal RMSE threshold θ was found to be 0.011 for dataset 1. The RMSE threshold for sparse was obtained by manual check of spectra in broadband view. The preclassification of the sparse spectra gave exactly the same results as for the broadband spectra: The same 21 spectra were classified as water in both the broadband and sparse datasets. Here it is important to mention that different θ thresholds for broadband spectra and seven WNs were due to difference in a number of spectral channels. In addition to the proposed MSC preclassification approach in this study, the OPUS quality test was performed on the broadband and seven WNs; however, due to the strict criteria set by OPUS, all the spectra were identified as low-quality spectra.

## 3. Discussions

### FTIR Spectra

Spectral data quality assessment is always challenging and done with care since the large reduction of a dataset can usually be detrimental for the establishment of classification and/or regression models. This is particularly challenging in medical studies, which often have a limited number of measured samples. A method to select high quality spectra is desirable and improves further analysis of the data to make reasonable conclusions and discoveries [41,44,45]. This study utilized knee cartilage FTIR spectra from human and bovine samples. The morphology and chemistry of damaged and healthy cartilage is different, resulting in detectable spectral differences. However, these differences are detectable only if high-quality spectra with strong analyte signals are acquired. If the ATR crystal is not in good contact with the sample surface due to insufficient pressure, the resulting spectrum can be of high quality (due to good contact with the surrounding water/PBS/synovial fluid) but with little or no absorbance signals from the analyte (cartilage).

Therefore, it is important to correctly identify water-like spectra, and remove them before further analysis since they do not contain any diagnostic value. This task of separating analyte-poor (water) spectra from analyte-rich (cartilage) spectra is relatively simple when applied on a broadband spectral dataset, and can be done quickly by visual inspection. However, if one relies on visual inspection on sparse datasets, where only few spectral bands are present, this task becomes difficult or nearly impossible.

In this study, we considered both the broadband FTIR dataset as well as the sparse dataset based on selecting seven WNs from the broadband dataset. The seven WNs were selected from the broadband spectra based on cartilage-specific absorbance, 850 cm${}^{-1}$), 1745 cm${}^{-1}$, 1620 cm${}^{-1}$, 1560 cm${}^{-1}$, 1210 cm${}^{-1}$, 1080 cm${}^{-1}$, and at 1800 cm${}^{-1}$. While the MSC model with baseline and multiplicative parameters can be expanded with higher terms such as linear and quadratic terms (the so-called extended multiplicative scatter correction (EMSC) model) [37,46], this was not done here due to the limitation with degrees of freedom.

Different options of the preclassification can in general be tested. When water spectra are to be separated from spectra containing cartilage signals, one approach would be to use water as a reference spectrum in MSC, while another approach is to use a representative cartilage spectrum. We tested both approaches and observed that using a cartilage spectrum was not as good as using a water spectrum. The method which is based on MSC, where a water spectrum was used as a reference spectrum, worked very well and allowed the separation both for the broadband dataset and the sparse seven WN dataset. The same or almost the same spectra were classified as analyte-poor spectra for both the broadband and sparse data preclassification, demonstrating that the method is consistent and robust.

In addition, we tested different water spectra: Those visually identified as water spectra in the datasets, and spectra obtained by measurements of pure water. The results were very similar, showing that irrespective of water spectrum used, we could obtain good preclassification.

When using cartilage as reference spectrum, the bad separation of water from cartilage spectra was probably caused by large differences between spectra of cartilage samples [6,36]. Distinct cartilage information can be seen in Figure 1. Contrary to variations in cartilage spectra, the water spectra have relatively small variation due to a much simpler absorbance pattern, containing just two peaks in the fingerprint region, Figure 3.

In this study, PCA was used to find and justify the selection of optimum RMSE threshold value. In Figure 3b,e,g, PCA scores are presented, and we can clearly observe how the separation between the two groups of spectra (water and cartilage spectra) changes with different RMSE threshold values. Similarly, the signal-to-noise ratio provides clear separation of water and cartilage spectra. The signal-to-noise ratio was calculated by Signal/Noise.

The PCA scatter plot then clearly justified optimization of the RMSE threshold values (θ), and showed the optimal boundary between the group of water and the group of cartilage spectra. As expected, the optimal parameter θ value was dataset dependent. While finding the optimal RMSE threshold value θ by an automatic procedure would be desirable, this was outside the scope of this study. For comparison with the here-presented MSC preclassification model, we also conducted an OPUS quality test. However, this quality test was unable to identify any quality spectra, indicating that the thresholds of OPUS test are too strict to be used universally. This is expected considering that the test was developed and optimized for entirely different types of samples, namely bacteria.

## 4. Materials and Methods

### 4.1. Spectral Data

In this study, we used two sets of articular cartilage samples from cadavers and carcasses from human and bovine samples, respectively. The broadband dataset of 282 samples from nine human cadavers was recorded by two different Alpha II FTIR spectrometers (Bruker Optics, Ettlingen, Germany), equipped with a globar MIR source and a deuterated triglyceride sulphate (DTGS) detector, each coupled to a single reflection diamond attenuated total reflection (ATR) accessory Platinum (Bruker Optics, Ettlingen, Germany). The IR spectra recorded with a total of 128 scans were averaged and there was a spectral resolution of 2 cm${}^{-1}$, a digital spacing of 1.0292 cm${}^{-1}$, over the range of 400–4000 cm${}^{-1}$, using the horizontal ATR diamond prism with 45° angle of incidence. The OPUS software (Bruker Optik GmbH, Ettlingen, Germany) was used for data acquisition and instrument control. The data generated by these two instruments will be called set 1 and set 2 throughout the study. The samples were hydrated articular cartilage sections from both knee joints. Each sample was prepared by drilling cylindrical 4 mm osteochondral plugs with a dental drill from central locations of femoral, tibial, and patellar cartilage. From each knee, multiple samples were extracted. The sample plugs were immersed in phosphate-buffered saline (PBS) and frozen to −80 °C for storage. Each sample was measured in triplicate, resulting in 846 spectra for set 1, and 815 for set 2. A third dataset, which we call set 3 throughout the study, was recorded by a Thermo Fischer Nicolet iS50 (Thermo Nicolet Corporation, Madison, WI, USA), equipped with a globar MIR source and a liquid nitrogen cooled mercury cadmium telluride (MCT) detector for bovine samples. The IR spectra were recorded with a total of 64 scans and spectral resolution of 2 cm${}^{-1}$, and a digital spacing of 0.2411 cm${}^{-1}$, over the range 400–4000 cm${}^{-1}$, using a custom-made ATR probe (Art Photonics GmbH, Berlin, Germany). The OMNIC software (Thermo Nicolet Corporation, Madison, WI, USA) was used for data acquisition and instrument control. Set 3 consisted of measurements of 132 samples, distributed across 10 bovine cadaver knees, with one knee per cadaver. Each sample was measured in triplicate, resulting in a total of 396 spectra.

To simulate sparse spectra of MIRACLE prototype device, seven WNs representing highly relevant peaks for cartilage quality determination were pre-selected. The seven WNs were selected from the broadband spectra based on cartilage-specific absorbance, as the absorbance at wavenumber 850 cm${}^{-1}$ is a band related to librations of water in the cartilage and synovial fluid. The absorbance at wavenumber 1745 cm${}^{-1}$ corresponds to the C=O stretching vibration of lipids present in the cartilage and synovial fluid. The absorbance at wavenumber 1620 cm${}^{-1}$ corresponds to amide I due to C=O stretching vibration of collagen, and the absorbance at wavenumber 1560 cm${}^{-1}$ corresponds to the amide II vibration associated with C-N-H stretching and bending vibration of collagen. The absorbance at wavenumber 1210 cm${}^{-1}$ corresponds to the O=C-N-H stretching and bending vibration (amide III) of collagen, and lastly, the absorbance at wavenumber 1080 cm${}^{-1}$ corresponds to the C-O stretching vibration of carbohydrate residues in collagen and proteoglycans [47]. For baseline estimation, the absorbance at the wavelength at 1800 cm${}^{-1}$ was chosen. The suggested preclassification approach in this study is based on multiplicative signal correction (MSC) that allows separation of water spectra from cartilage spectra. To the best of our knowledge, there are no similar studies available where the MSC model was used for preclassification of infrared spectral data.

### 4.2. Annotation of Broadband Spectra for Water, Cartilage, and Low Signal Spectra

In this study two methods were used to annotate the infrared spectra. The first method was based on the OPUS quality test, utilizing the signal-to-noise ratio. The absorbance signal (Signal) was calculated in the range 920–1200 cm${}^{-1}$ as the difference between the maximum and the minimum of the first derivative in the range. The second parameter (Noise) was calculated in the range 2000–2100 cm${}^{-1}$ as the difference between the maximum and the minimum of the first derivative in the range. The signal-to-noise ratio was calculated by dividing Signal/Noise. The second method was based on Principle Component Analysis (PCA) of the spectral data. PCA score plots were used to visually identify possible clusters of water/analyte-poor spectra and cartilage spectra of broadband data. Both methods require finding a threshold to separate cartilage from water spectra. These two methods were used to annotate the spectra as water and cartilage. The results of the new preclassification method based on MSC proposed in this study were then compared to the annotated spectra.

## 5. Theory

In this study, we suggest to perform a preclassification of the spectra to separate spectra of water from cartilage spectra by the multiplicative signal correction method (MSC). MSC was introduced in the 1980s to separate scatter effects from chemical information in near-infrared spectroscopy [48]. MSC has been further developed and widely used for removing various physical and unwanted chemical variations from spectra [37,48,49,50,51] as well as for quality testing of the spectra and background removal in hyperspectral image analysis [41,52]. The MSC model can be written as follows (Equation (1)):

(1) $$Z_{}\left(\tilde{\nu }\right)=a+b\cdot Z_{ref}\left(\tilde{\nu }\right)+\epsilon$$

where $Z_{ref}\left(\tilde{\nu }\right)$ is a reference spectrum, *b* is a multiplicative scaling parameter, the parameter *a* captures additive baseline variations, while the term ϵ is a residual capturing unmodelled variations. Thus, in the MSC model, every measured spectrum $Z(\tilde{\nu })$ is modelled around a reference spectrum $Z_{ref}$ using a multiplicative factor *b* and a baseline factor *a*. In an ideal situation, the unmodelled effects ϵ contain the chemical variations (which are usually of main interest) [48], while all physical effects are modelled by the MSC model. The algorithm proposed in this study suggests the use of water spectrum as a reference spectrum, while the residual spectrum ϵ is to be used to determine how close a measured spectrum is to the water spectrum. Thus, the MSC model used for the preclassification can be written as follows (Equation (2)):

(2) $$Z\left(\tilde{\nu }\right)=a+b\cdot Z_{water}\left(\tilde{\nu }\right)+\epsilon$$

As metrics of the dissimilarity to the reference spectrum, we use the root mean squared error (RMSE) given by Equation (3):

(3) $$RMSE\left(\epsilon \right)=\sqrt{\frac{1}{n}\sum_{i}\epsilon_{i}^{2}}$$

To decide if a measured spectrum is a water spectrum or not, we need to set a threshold θ for the RMSE. When a threshold is set, RMSE(ϵ) is calculated for each spectrum and the spectrum can be either identified as water or cartilage, Equation (4):

(4) $$\begin{array}{c} \mathrm{if}\;RMSE\left(\epsilon \right)<=\theta ,\mathrm{then}\;Z\left(\tilde{\nu }\right)\;\mathrm{is}\;\mathrm{water};\\ \mathrm{if}\;RMSE\left(\epsilon \right)>\theta ,\mathrm{then}\;Z\left(\tilde{\nu }\right)\;\mathrm{is}\;\mathrm{cartilage}. \end{array}$$

To determine the threshold θ for broadband and sparse data, we used visual inspection of the broadband spectra. The MSC approach was implemented on broadband and seven WN spectra of datasets 1, 2, and 3. The MSC preclassification approach, PCA, and signal-to-noise ratio were performed by algorithms developed in house, and with open-source algorithms in Matlab, R2020a (The Mathworks Inc., Natick, MA, USA).

## 6. Conclusions

In ATR infrared spectroscopy of cartilage in medical applications, measured spectra may suffer from strong water signals because of low contact with the cartilage. To make a transition of the technology from the lab to the hospital possible, an automated preclassification of spectra into quality cartilage spectra and spectra with strong water signal is needed.

The study presented a preclassification algorithm based on the multiplicative signal correction (MSC) method to separate water spectra from spectra containing cartilage signals for broadband and sparse data. In the algorithm, a pure water spectrum was used as reference in the MSC model, and root mean squared error (RMSE) values were used to classify the spectra as analyte rich (i.e., “cartilage-like” spectra) or analyte poor (i.e., “water-like” spectra). The RMSE threshold parameter, used for the classification, needs to be optimized, and is dataset dependent.

With the optimal RMSE threshold the method works well both for the broadband spectra and the sparse data. The results of the preclassification were similar both for the broadband spectra and sparse spectra of the same dataset; the same or nearly the same spectra were removed in both cases. The method is quite general and can be used in other applications for spectral preclassification, where other types of analyte and matrix constituents are present.

## Data Availability

Data that support the findings of this study not available.

## Supplementary Materials

The following supporting information can be downloaded at: https://www.mdpi.com/article/10.3390/molecules27072298/s1, Figure S1: Visual presentation of broadband spectrum after using MSC preclassification approach (Collagen as reference spectrum), (a) Water/Analyte-poor Spectra (b) Cartilage/Analyte-rich Spectra; Figure S2: Visual presentation of Human 12 (set 2) broadband spectra after using MSC preclassification approach, (a,d,g) Water/Analyte-poor Spectra (c,f,i) Cartilage/Analyte-rich Spectra and (b,e,h) are PCA scores obtained by different RMSE thresholds, 0.1 for (b), 0.2 for (c) and 0.22 for (h). c and w in PCA scores correspond to cartilage and water spectrum; Figure S3: Visual presentation of Bovine (set 3) broadband spectra after using MSC preclassification approach, (a,d,g)Water/Analyte-poor Spectra (c,f,i) Cartilage/Analyte-rich Spectra and (b,e,h) are PCA scores obtained by different RMSE thresholds, 0.5 for (b), 0.6 for (c) and 0.12 for (h). c and w in PCA scores correspond to cartilage and water spectrum.

## Sample Availability

Samples of the compounds are not available from the authors.
