# Supplementary material for: Preclassification of Broadband and Sparse Infrared Data by Multiplicative Signal Correction Approach
Source: Molecules. 2022 Apr 1;27(7):2298. doi: 10.3390/molecules27072298 (PMC9000438; doi:10.3390/molecules27072298)
Supplement: Supplementary file 1 [file molecules-27-02298-s001.zip › molecules-1621374-supplementary.pdf]

molecules

MDPI

Article

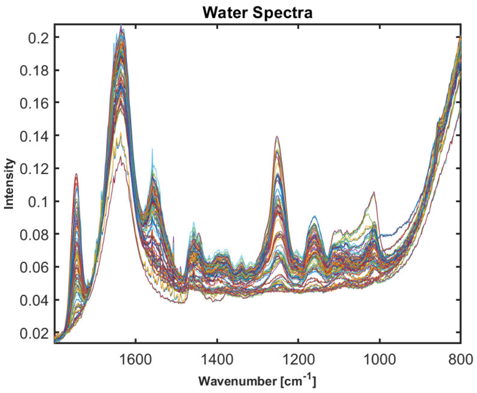

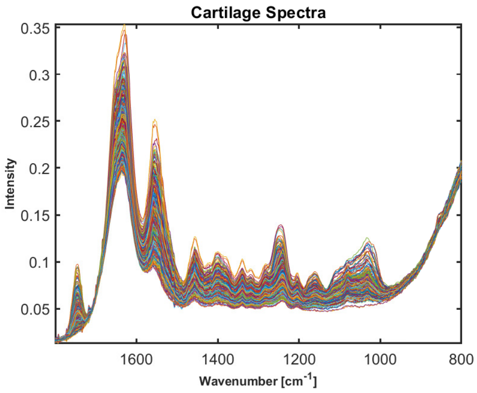

(**a**) Analyte-poor Spectra (**b**) Analyte-rich Spectra

Figure S1. Visual presentation of broadband spectrum after using MSC preclassification approach (Collagen as reference spectrum), (**a**) Water/Analyte-poor Spectra (**b**) Cartilage/Analyte-rich Spectra.

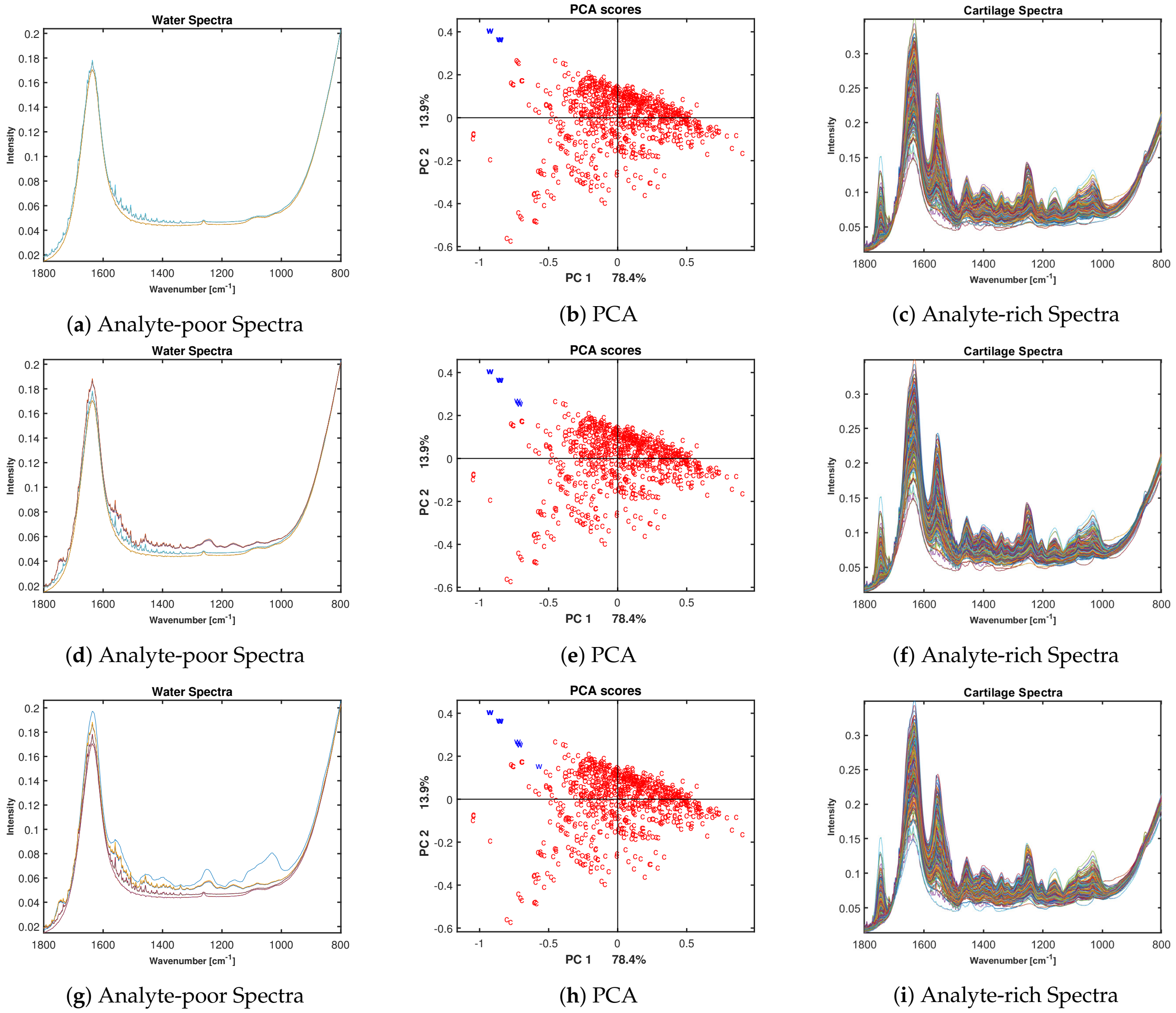

(**a**) Analyte-poor Spectra (**b**) PCA (**c**) Analyte-rich Spectra

(**d**) Analyte-poor Spectra (**e**) PCA (**f**) Analyte-rich Spectra

(**g**) Analyte-poor Spectra (**h**) PCA (**i**) Analyte-rich Spectra

Figure S2. Visual presentation of Human 12 (set 2) broadband spectra after using MSC preclassification approach, (**a**,**d**,**g**) Water/Analyte-poor Spectra (**c**,**f**,**i**) Cartilage/Analyte-rich Spectra and (**b**,**e**,**h**) are PCA scores obtained by different *RMSE* thresholds, 0.1 for (**b**), 0.2 for (**c**) and 0.22 for (**h**). c and w in PCA scores correspond to cartilage and water spectrum.

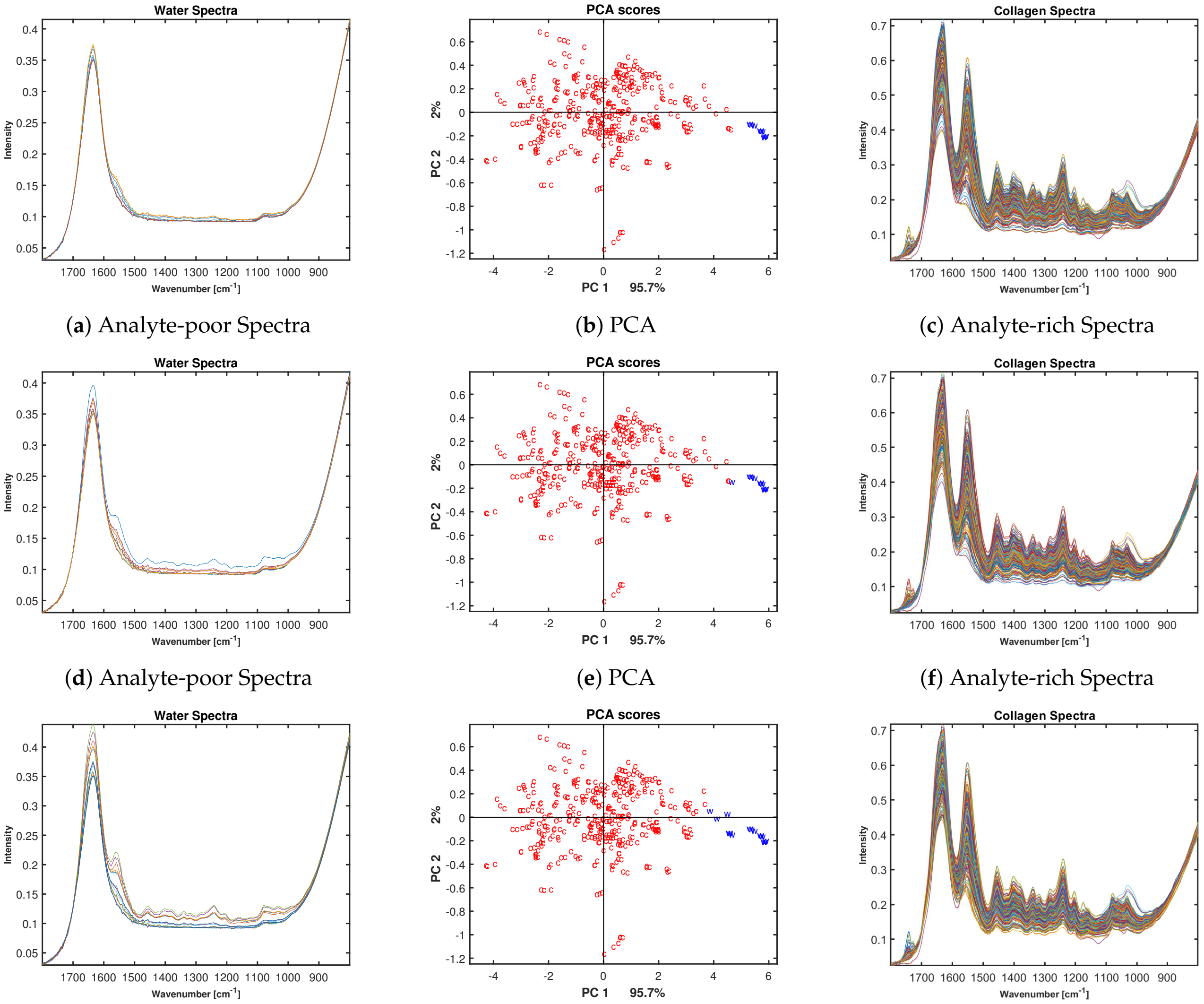

(**a**) Analyte-poor Spectra (**b**) PCA (**c**) Analyte-rich Spectra

(**d**) Analyte-poor Spectra (**e**) PCA (**f**) Analyte-rich Spectra

(**g**) Analyte-poor Spectra (**h**) PCA (**i**) Analyte-rich Spectra

Figure S3. Visual presentation of Bovine (set 3) broadband spectra after using MSC preclassification approach, (**a**,**d**,**g**) Water/Analyte-poor Spectra (**c**,**f**,**i**) Cartilage/Analyte-rich Spectra and (**b**,**e**,**h**) are PCA scores obtained by different *RMSE* thresholds, 0.5 for (**b**), 0.6 for (**c**) and 0.12 for (**h**). c and w in PCA scores correspond to cartilage and water spectrum.
